# Evaluating the effectiveness of enhanced family planning education on knowledge and use of family planning in fishing communities of Lake Victoria in Uganda: a randomized controlled trial

**DOI:** 10.1186/s12913-022-07898-3

**Published:** 2022-04-14

**Authors:** Annet Nanvubya, Rhoda K. Wanyenze, Andrew Abaasa, Teddy Nakaweesa, Juliet Mpendo, Barbarah Kawoozo, Francis Matovu, Sarah Nabukalu, Geoffrey Omoding, Jed Kaweesi, John Ndugga, Bernard Bagaya, Kundai Chinyenze, Matt A. Price, Jean Pierre Van Geertruyden

**Affiliations:** 1 UVRI-IAVI HIV Vaccine Program, Entebbe, Uganda; 2 Global Health Institute, University of Antwerp, Antwerp, Belgium; 3 School of Public Health, Makerere University College of Health Sciences, Kampala, Uganda; 4 MRC/UVRI & LSHTM Uganda Research Unit, Entebbe, Uganda; 5 Uganda Virus Research Institute, Entebbe, Uganda; 6 International AIDS Vaccine Initiative, New York, USA

**Keywords:** Family planning, Knowledge, Use, Fishing communities

## Abstract

**Introduction:**

Family planning knowledge is poor and use is low in Ugandan fishing communities. We compared the effectiveness of enhanced family planning (FP) education with routine counselling on FP knowledge and use.

**Methods:**

Individuals aged 15–49 years were randomly assigned to intervention or control arm. The intervention constituted enhanced FP education based on a simplified handout extracted from the WHO FP guidance tool called, “Family planning: A global handbook for FP providers” which participants took home for additional reading. The control arm constituted FP counselling following Uganda Ministry of Health guidelines. FP knowledge score and contraceptive prevalence rate (CPR) were compared between trial arms at baseline and at 12 months. Negative binomial regression models were used to estimate the effect of the intervention on FP knowledge and use.

**Results:**

Overall, 1410 participants were screened to enrol 1004 (502 per study arm, 48.5% women). Subsequently, 384 (76.5%) and 383 (76.3%) completed the 12 months’ follow-up in the intervention and control arms respectively. At baseline, a median FP knowledge score of 8 and a < 70% FP knowledge score was observed for all participants with a CPR of 36.8%. At month-12, the median FP knowledge score improved in both arms, higher in the intervention arm than the control arm (46 vs 30; *p* < 0.001). In the intervention arm, 304 (79.2%) had a score of ≥70 compared with 21 (5.5%) in the control arm (*p* < 0.001). In the negative binomial regression model, the change in FP knowledge score was 47% higher in the intervention arm than in the control arm (score ratio: 1.47, 95%CI: 1. 43-1.51, *p* < 0.001). The change in CPR was 16% higher in the intervention arm than in the control arm (Prevalence ratio: 1.16, 95%CI: 1.01-1.34, *p* < 0.040).

**Interpretation:**

Enhanced FP education using a simplified FP education handout was more effective in increasing FP knowledge and use compared to routine FP counselling for people living in fishing communities. Innovative FP education interventions are recommended for improving FP knowledge and optimizing uptake in remote-rural settings where literacy levels are low.

**Trial registration:**

The study was registered by the Pan African Clinical Trial Registry on 03 July 2021 with a Trial Registration Number PACTR202107891858045. “Retrospectively registered”.

## Introduction

Family planning (FP) use is associated with good health and economic outcomes [1]. However, there are still gaps in FP uptake globally especially in resource limited settings [2]. To attain the sustainable development goals (SDGs), there are global efforts to improve knowledge, access, availability and use of FP. Despite these efforts, Contraceptive Prevalence Rate (CPR) remains low in many developing countries in Africa [3]. The factors contributing to the low contraceptive uptake vary in different countries depending on their social, economic, environmental, and political status. Like elsewhere in the world, Uganda is committed to ensuring universal health coverage and access, and as such, Uganda has invested in initiatives like training of more medical personnel, building and equipping health facilities. Nevertheless, Uganda remains one of the countries with the highest maternal, new-born and child mortality rates [4–6] and the lowest CPR globally [7]. To improve maternal and infant mortality, ensuring a good contraceptive prevalence rate (CPR) in addition to universal health coverage remains critical.

According to the 2016 demographic and health survey for Uganda, 39% of married women were reported to be using FP while 28% had an unmet need for FP [8]. In this survey, FP constituted a conscious effort by a couple to limit or space the number of children they have through the use of contraceptive methods. Women who wanted to postpone their next birth for 2 or more years or who wanted to stop childbearing altogether but were not using a contraceptive method were reported to have an unmet need for FP. In fulfilment of the FP2020 targets, Uganda committed to reduce unmet need for family planning from 40 to 10% by 2022 [9]. To increase contraceptive uptake, the ministry of health and other implementing partners made deliberate efforts to sensitize and provide FP services across the country. Despite these efforts, the CPR is persistently low in the country, particularly so in Uganda’s underserved fishing communities [10–12]. Fishing communities in Uganda have poor access to health services and are characterised by high rates of sexually transmitted infections like HIV and syphilis [13]. Improvement of reproductive health services in the fishing communities is critical because they contribute greatly to the country’s gross domestic product and have a right to good health.

While the concept of FP is almost universally known in Uganda, there are still misconceptions about its effects particularly among marginalized populations [14]. Our previous findings showed that poor knowledge of FP was associated with low FP uptake in fishing communities [15]. Good knowledge of a wide range of FP methods enables informed and timely choices among people in need of FP services [16–20]. Reproductive health service centres in Ugandan fishing communities tend to be scarce and haphazard which limits people’s choices. Besides, these centres also tend to offer a limited range of options [11].

Ugandan fishing communities also tend to be characterized by low literacy levels [21]. Low literacy is associated with poor comprehension of mechanisms of action, eligibility criteria and adverse effects of contraceptive methods [22]. The low literacy levels could also explain the myths and misconceptions about FP that still exist in fishing communities [23]. Recent findings from a study that was conducted in fishing communities showed that the effectiveness of some FP methods was doubted and some side effects were exaggerated or even confused with other causes of ill health [11]. This underscores a need for comprehensive contraceptive education to improve knowledge.

As in other rural settings in Uganda, religious and cultural beliefs in favour of large families negatively impact FP use [24, 25]. To address the negative religious and cultural beliefs in such communities, innovative and creative education mechanisms that suit their context may be required while delivering reproductive health information. We conducted a randomized controlled trial to assess the effectiveness of enhanced family planning education using a simplified education tool on FP knowledge and use.

## Methodology

### Study setting and population

Participants were recruited from two fishing communities on Lake Victoria in Uganda: Kigungu landing site on the mainland and Nsazi which is an island community. The two sites were selected based on their location (one being an island and the other a mainland site) and their population size as they are among the most densely populated fishing communities along Lake Victoria [26]. Kigungu has a population of approximately 30,000 people while Nsazi has a population of up-to 8000 people. The study targeted community residents where a resident was considered as anyone who stayed or was employed in the study area for at least 6 months. Like most fishing communities in Uganda, these communities have a high presence of bars, multiple sexual partnerships with limited access to essential healthcare services [27–30]. They have few government and private health facilities with the following FP methods available at the facilities; pills, condoms, injectable hormones, IUDs, implants, and emergency pills.

### Study design

The study employed an open-label, randomized control trial design with one intervention and control arm. The study intervention constituted FP education using a simplified handout that was extracted from the WHO FP guidance tool called “Family planning: A global handbook for providers” [31]. The handbook contains medical information that helps health care providers deliver FP methods appropriately and effectively to clients. It also contains tools for counselling and education on different FP methods. It covers related health issues that may arise in the context of FP. It provides specific guidance on 20 FP methods, their doses and contraindications and addresses many of clients’ different needs, from correcting misunderstandings to managing side effects. The handout was designed to have simple short sentences with pictures of the FP methods and it was translated into Luganda, the local language, by a certified translator. Special considerations for the literacy level of fishing community residents were made by the translator through use of commonly known illustrations. To ensure the appropriateness, reliability and validity of the handout, it was piloted (along with the other FP materials) among 30 individuals in a non-study fishing community before it was used. Study staff were trained to administer the handout prior to the pilot. Participants in the control arm received routine FP counselling as per the Ministry of Health (MoH) FP counselling guide. The MoH FP counselling guide constitutes information about 12 FP methods and it is in the English language. The information is presented in form of sentences with no pictures. The sentences are read verbatim to clients or sometimes translated by the health provider (based on their interpretation) for clients who may be illiterate. No reading materials are given to clients for further reading. Participants in both arms were offered FP methods of their choice after confirmation of eligibility. Eligible participants were followed up at months 6 and 12.

### Sample size determination

**Fig. 1:**
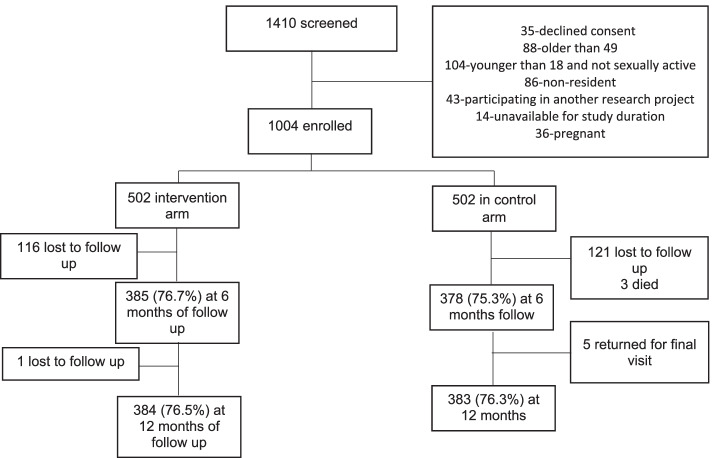
Recruitment characteristics in a trial that evaluated the effectiveness of enhanced FP education in Ugandan fishing communities

To determine the sample size, we assumed a baseline CPR of 35.2% and an effect difference of 10% [12]. To obtain 80% power, a significance level of 5% and a 25% loss to follow up were considered [29]. A sample size of 1004 participants with 502 participants per study arm was calculated (Fig. 1).

### Inclusion and exclusion criteria

Participants aged 15–49 years, both men and women, who were willing to give their written consent or assent for participation and comply with study procedures were eligible to participate in the study. Participants below 18 years were included only if they were sexually active. All participants pregnant at screening or participating in other research studies were excluded from the study.

### Randomization procedure

The eligible participants provided written informed consent or assent to participate in the trial and were randomly assigned to either intervention or control arm based on a predetermined randomization schedule which was computer-generated by the data manager using STATA (Version 15, College Station, TX). Individual randomization was done, and once the required sample size was accrued, enrolment ceased. Study staff and participants were not blinded in terms of who was in the intervention arm or the control arm.

### Data collection

Training of the study team on the study procedures and how to use the data collection tools was done prior to commencement of the study. Study questionnaires were pretested to check the appropriateness of translation, the skip actions and to address any clarifying questions. Any necessary modifications were made prior to study start. Five experienced and well-trained interviewers collected data on social demographic characteristics, FP knowledge and other reproductive health aspects using anonymized semi-structured questionnaires.

### Measures

The two major outcomes of interest were 1) knowledge of and 2) use of FP, collected at baseline and again at the end of 12 months. Participants were asked if they had heard of or knew any FP method. Those who responded in the affirmative were then asked to list, unprompted, which methods they knew or were aware of. Participants were asked to mention the sources of the FP information known by them. Participants were further asked if they were using any FP method. Those who responded in the affirmative were asked to mention the method they were using, how they had heard about the method, and whose decision it was to use FP. They were also asked if they had used condoms in the past 12 months. Those who were not using FP were asked to give reasons why.

To assess their FP knowledge, participants were asked a series of 64 questions about FP and the different methods [15]. Questions were asked about the following FP methods; pills, injectable hormonal methods, implants, emergency contraceptive pills, intra-uterine device, vasectomy, tubal ligation, condoms, spermicides, diaphragm, withdraw, breast feeding (lactation amenorrhea), calendar, moon beads, periodic abstinence, foam/jelly, herbs and dermal patch. The questions were asked in regard to eligibility criteria for FP, mechanisms and duration of action, routes of administration, adverse effects, how these adverse effects can be managed and what needs to be done in case of a missed dose or if a replacement is required, other benefits of FP besides contraception and FP use in the context of HIV. Questions that were correctly answered were scored 1 while those that were wrongly answered or where the participant said they didn’t know the answer were scored 0 [15]. Knowledge grade was categorized into good or poor knowledge based on the percentage score. Participants’ use or non-use of FP was set as a binary outcome variable. We defined FP use as a participant reporting self or partner use of any (one or more) of the FP methods. All methods were given out in accordance with relevant guidelines and regulations.

### Data management and analysis

Double data entry was done in Microsoft Access database and data were managed and analysed using STATA. During analysis, we compared participants’ baseline characteristics between control and intervention arms using counts and percentages. We estimated both baseline and end of follow up FP knowledge score as the total of a participant’s score on the 64 questions using means with standard deviation and medians with range. We further expressed FP knowledge score as a percentage i.e. total score divided by 64 multiplied by 100. We compared the end of follow up data to the baseline data. We established a dichotomous outcome of “good” vs “poor” knowledge that allowed adequate numbers of outcomes to assess impact of the intervention, settling on a cut off of ≥70% correct answers as “good”. We fitted a negative binomial regression model controlling for baseline score to estimate the effect of the intervention on the FP knowledge. We preferred negative binomial regression models because of the dispersion in the count data and the more precise confidence intervals compared to those from Poisson regression models. We further fitted a log-binomial regression model controlling for baseline knowledge score to estimate the effect of the intervention on FP use. We preferred log-binomial because of the high proportion using FP as Odds ratios from logistic regression would over estimate prevalence ratios. We compared the CPR at baseline and at 12 months.

We estimated baseline HIV and Syphilis prevalence as the number that tested positive divided by total number tested expressed as a percentage. HIV and syphilis incidence were also calculated at the end of follow up as the number who became positive divided by person years at risk (PYAR) expressed as per 100 PYAR.

## Results

Between February and November 2017, a total of 1410 participants were screened to enrol 1004 participants with 502 in each arm (Fig. 1). Detailed demographic characteristics of participants in both study arms are presented in Table 1. Overall, 48.5% of participants were women, and this didn’t vary by study arm. The mean age of participants was approximately 28.0 years in both study arms. A total of 767 (76%) participants completed follow-up and were assessed with no significant variation by study arm (Fig. 1).

**Table 1:** Baseline demographic characteristics of participants in a trial that evaluated the effectiveness of enhanced FP education in Ugandan fishing communities

Characteristic	Intervention arm ***n*** = 502 (%)	Control arm ***n*** = 502 (%)
Mean Age (SD)	27.7 (7.1)	27.8 (7.3)
Median Age (IQR)	26 (22-32)	26 (22-32)
**Age group (Years)**		
15-29	312 (62.1)	324 (64.5)
30-39	157 (31.3)	133 (26.5)
40+	33 (6.6)	45 (9.0)
**Sex**		
Male	244 (48.6)	273 (54.4)
Female	258 (51.4)	229 (45.6)
**Study village**		
Kigungu	402 (80.1)	402 (80.1)
Nsazi	100 (19.9)	100 (19.9)
**Tribe**		
Muganda	224 (44.6)	222 (44.2)
Munyankole	45 (9.0)	47 (9.4)
Musoga	41 (8.2)	28 (5.6)
Mukiga	12 (2.4)	12 (2.4)
Munyarwanda	46 (9.2)	41 (8.2)
Other^a^	134 (26.6)	152 (30.2)
**Occupation**		
Farming	14 (2.8)	13 (2.6)
Fishing/Fishing related	182 (36.2)	180 (35.8)
Hotel/Bar/Hair salon	42 (8.4)	26 (5.2)
Trade/business	23 (4.6)	16 (3.2)
House wife	39 (7.8)	42 (8.4)
Otherβ	202 (40.2)	225 (44.8)
**Religion**		
Catholic	215 (42.9)	204 (40.7)
Protestant/Anglican	115 (22.9)	119 (23.7)
Muslim	88 (17.5)	70 (13.9)
Born again Christian	76 (15.1)	95 (18.9)
Other^b^	8 (1.6)	14 (2.8)
**Highest Education level**		
No formal education	32 (6.4)	26 (5.2)
Primary level	232 (46.2)	254 (50.6)
Secondary level	196 (39.0)	181 (36.1)
Tertiary/University	42 (8.4)	41 (8.1)
**Marital status**		
Single/Never married	126 (25.1)	124 (24.7)
Married	293 (58.4)	288 (57.4)
Single/Ever married	83 (16.5)	90 (17.9)
**Duration of stay (years) in community**		
0-1	186 (37.1)	173 (34.5)
2-4	131 (26.1)	148 (29.4)
5+	185 (36.8)	181 (36.1)
**Having multiple sexual partners in past 12 months**		
No(< 2 partners)	333 (66.3)	321 (63.9)
Yes(> = 2 partners)	90 (17.9)	79 (15.8)
Not specified	79 (15.8)	102 (20.3)
**Currently in a sexual Relationship?**		
Yes	423 (84.3)	400 (79.7)
No	79 (15.7)	102 (20.3)
**FP Awareness**		
Yes	477 (95.0)	476 (94.8)
No	25 (5.0)	26 (5.2)

^a^Mugisu, Itesot, Non-Uganda, β Sex worker, Teacher, Security personnel and others

^b^Pentecostal/ Born again, Traditional African, No religion

### Awareness of family planning methods

Participants were asked if they were aware of any FP method and to mention (Unprompted) which methods they were aware of. Most participants (95%) were aware of at least one FP method with modern FP methods including pills, injectable hormones and implants being more popular than others at baseline (Table 2). Only a few participants were aware of permanent methods (Tubal ligation and Vasectomy) at baseline. After 12 months of follow-up, the number of participants who were aware of FP methods and the different methods of FP known increased in both arms, however a greater number of persons in the intervention arm were able to name more types of FP. More participants in the intervention arm were aware of spermicides, periodic abstinence, calendar method, IUD, tubal ligation, vasectomy, implants, rhythm/withdraw method, diaphragm, dermal patch, emergency pill, foam/Jelly and moon beads than those in the control arm (Table 2).

**Table 2:** Awareness of FP methods in a trial that evaluated the effectiveness of enhanced FP education in Ugandan fishing communities

Variable	At Baseline			After 12 Months		
	Intervention arm *N* = 502 *n* (col %)	Control arm *N* = 502 *n* (col %)	*p*-value	Intervention arm *N* = 384 n (col %)	Control arm *N* = 383 n (col %)	*p*-value
**Aware of FP method**						
Yes	477 (95.0)	476 (94.8)	0.886	384 (100)	383 (100)	–
Unable to list any FP method	25 (5.0)	26 (5.2)		0 (0)	0 (0)	
Pills	365 (76.5	370 (77.7)	0.656	380 (99)	376 (98.2)	0.360
Condom	249 (52.2)	247 (51.9)	0.924	371 (96.6)	362 (94.5)	0.158
Injectable hormones	352 (73.8)	363 (76.3)	0.379	365 (95.3)	376 (97.9)	0.045
Spermicide	9 (1.9)	14 (2.9)	0.289	105 (27.3)	70 (18.3)	0.003
Periodic Abstinence	24 (5.0)	37 (7.8)	0.084	204 (53.1)	143 (37.3)	< 0.001
Calendar	21 (4.4)	19 (4.0)	0.752	211 (54.9)	145 (37.9)	< 0.001
IUD/Coil	233 (48.8)	227 (47.7)	0.721	339 (88.3)	320 (83.6)	0.060
Breast-feeding/ Lactation Amenorrhea	15 (3.2)	15 (3.1)	0.995	243 (63.3)	196 (51.2)	0.001
Tubal ligation	36 (7.6)	36 (7.6)	0.993	301 (78.4)	253 (66.1)	< 0.001
Vasectomy	44 (9.2)	44 (9.3)	0.992	310 (80.7)	265 (69.2)	< 0.001
Implants/Norplant	286 (60.0)	275 (57.8)	0.493	359 (93.5)	336 (87.7)	0.006
Rhythm/Withdraw method	58 (12.2)	63 (13.2)	0.635	297 (77.3)	259 (67.6)	0.003
Diaphragm	1 (0.2)	3 (0.6)	0.315	50 (13.0)	30 (7.8)	0.019
Dermal Patch	0 (0)	0 (0%)	–	58 (15.1)	28 (7.3)	0.001
Emergency Pill	11 (2.3)	15 (3.2)	0.423	224 (58.3)	120 (31.3)	< 0.001
Moon beads	9 (1.9)	9 (1.9)	0.996	119 (31.0)	68 (17.8)	< 0.001
Foam/Jelly	1 (0.2)	2 (0.4)	0.562	47 (12.2)	17 (4.4)	< 0.001

### Family planning knowledge assessment

At baseline, the median FP knowledge score was 8 (range = 0-40) out of a max of 64 (12.5%). All participants scored less than 70% at baseline as shown in Table 3. After 12 months of follow up, the median FP knowledge score increased in both trial arms (both *p* < 0.001) but more so in the intervention arm (*p* < 0.001) where the median score was 46 (70.8%, range 25-57) compared to 30 (46.2%, range 10-55) in the control arm. The proportion of participants that scored 70% or more was 79.2% in the intervention arm compared to 5.5% in the control arm (*p* < 0.001).

**Table 3:** FP knowledge score in a trial that evaluated the effectiveness of enhanced FP education in Ugandan fishing communities

Time point	Measures of effect	Trial Arm		
		Intervention	Control	p-value
Baseline	Mean (SD)	10.4 (7.9)	10.2 (7.5)	0.73
	Median (range)	8 (0-40)	8 (0-37)	0.86
	Conditional variance	62.8	56.0	
	**Percent score**			
	Poor (< 70%)	384 (100%)	383 (100%)	
	Good (70% +)	0 (0.0%)	0 (0.0%)	
12 months	Mean (SD)	45.2 (5.0)	30.7 (7.1)	< 0.001
	Median (range)	46 (25-57)	30 (10-55)	< 0.001
	Conditional variance	24.8	49.4	
	**Percent score**			
	Poor (< 70%)	80 (20.8)	362 (94.5)	< 0.001
	Good (70% +)	304 (79.2)	21 (5.5)	

### Effect of intervention on family planning knowledge and use

Table 4 shows the results of the negative binomial regression for the FP knowledge score after 12 months of follow up between intervention and control arm after adjusting for the baseline FP knowledge score. At the end of the 12 months, the change in FP knowledge score in the intervention arm was 47% higher in the intervention arm than in the control arm (score ratio: 1.47, 95%CI: 1. 43-1.51, *p* < 0.001). In the negative binomial regression model, the change in CPR was 16% higher in the intervention arm than in the control arm (Prevalence ratio: 1.16, 95%CI: 1.01-1.34, *p* < 0.040).”

**Table 4:** Results of the negative/log binomial regression in a trial that evaluated the effectiveness of enhanced FP education in Ugandan fishing communities

Study arm	Adjusted Coefficient	95%CI	*p*-value	Adjusted SR/PR	95%CI	*p*-value
Intervention^c, *^	0.39	0.36-0.41	< 0.001	1.47^a^	1.43-1.51	< 0.001
Intervention^d, **^	0.15	0.06-0.29	0.040	1.16^b^	1.01-1.34	0.040

^a^SR-Score ratio

^b^PR-Prevalence ratio, *CI* Confidence interval

^c^FP Knowledge

^d^FP use (*analysis adjusted for baseline FP knowledge score, **analysis adjusted for baseline FP knowledge score)

### Family planning use by participants

The proportion using FP at baseline was approximately 37% and this did not vary by study arm as shown in Table 5. The most used methods in both study arms were condoms, injectable hormones, implants and pills. Just over a half (250; 53.1% in intervention and 243; 53.9% in control) reported condom use in the past 12 months. Nearly all participants reported either a Government hospital or clinic/health centre to be their source for the preferred FP method with very few indicating NGOs as their source for FP. While most participants reported that they jointly decided with their sexual partner to use FP, over a third in either study arm made independent or personal decisions to use FP. The most common reasons for not using FP included infrequent or no sex, fertility desire, economic constraints and side effects associated with use of FP.

**Table 5:** Baseline FP use and other characteristics regarding methods used in a trial that evaluated the effectiveness of enhanced FP education in Ugandan fishing communities

Variable	Intervention ***n*** = 502(%)	Control ***n*** = 502(%)
**FP use**		
Yes	188 (37.5)	181 (36.1)
No	314 (62.5)	321 (63.9)
**FP Methods Used**		
Pills	14 (7.2)	9 (4.8)
Condom	67 (34.4)	64 (33.9)
Injectable hormones	51 (26.2)	63 (33.3)
Implant/Norplant	48 (24.6)	38 (20.1)
Tubal-ligation	5 (2.6)	2 (1.1)
Rhythm/ Withdrawal	5 (2.6)	3 (1.6)
IUD/Coil	2 (1.0)	6 (3.2)
Other^a^	3 (1.5)	4 (2.1)
**Source of FP Method**		
Government hospital/clinic	173 (92.0)	166 (91.7)
Private hospital/clinic	8 (4.3)	11 (6.1)
NGOs	3 (1.6)	4 (2.2)
Ordinary Shop/weekly markets	3 (1.6)	0 (0.0)
Other^b^	1 (0.5)	0 (0.0)
**Decision to use FP**		
Mainly mine (respondent)	74 (39.4)	74 (40.9)
Mainly spouse/partner	9 (4.8)	17 (9.4)
Joint decision	105 (55.8)	89 (49.2)
Other^**c**^	0 (0.0)	1 (0.5)
**Condom use in past 12 Months**		
Yes	250 (53.1)	243 (53.9)
No	221 (46.9)	208 (46.1)
**Reasons for not using FP**		
Infrequent/no sex	80 (23.6)	94 (27.0)
Need for children/get pregnant	75 (22.1)	80 (23.0)
Economic constraints	90 (26.5)	75 (21.6)
Side effects of FP	45 (13.3)	38 (10.9)
Menstrual problems	15 (4.4)	10 (2.9)
Religion does not permit	14 (4.1)	14 (4.0)
Culture encourages more children	9 (2.7)	14 (4.0)
Spouse disapproved	7 (2.1)	13 (3.7)
Lack of sexual satisfaction	4 (1.2)	10 (2.9)

^a^Vasectomy, emergency pills, Breast feeding, Herbs, Calendar, Abstinence

^b^FP clinics, Medicine vendors

^c^friend/peer

### Effectiveness of enhanced FP education on other participant characteristics

Overall, FP use was higher after 12 months than at baseline in both arms (Table 6) and this effect was stronger in the intervention group. Similarly, statistical differences between baseline and at 12 months in both trial arms were observed for the source of FP services (*p* < 0.001) and discussion of FP with spouse (*p* = 0.04).

**Table 6:** Effectiveness of enhanced FP education on FP use and related participant characteristics in a randomized control trial in Ugandan fishing communities

Variable	Intervention ***n*** = 384(%)		***p***-value	Control ***n*** = 383(%)		***p***-value
	Baseline	After 12 months		Baseline	After 12 months	
**FP use**						
Yes	150 (39.1)	206 (53.6)	< 0.001	138 (36.0)	177 (46.2)	0.004
No	234 (60.9)	178 (46.4)		245 (64.0)	206 (53.8)	
**Choice of FP**						
Modern	144 (96.0)	195 (94.7)	0.62	133 (96.4	164 (92.7)	0.22
Natural	6 (4.0)	11 (5.3)		5 (3.6)	13 (7.3)	
**Opinion about FP effectiveness**						
Effective	150 (100)	206 (100)	na	136 (99.3)	177 (100)	0.44
Not effective	0 (0.0)	0 (0.0)		1 (0.7)	0 (0.0)	
**Source of FP services**						
Government hospital/clinic	55 (15.0)	2 (0.5)	< 0.001	72 (19.6)	8 (2.1)	< 0.001
Private hospital/clinic	132 (35.9)	32 (8.3)		132 (35.9)	44 (11.5)	
NGOs	77 (20.9)	91 (23.7)		68 (18.5)	144 (37.6)	
Pharmacy/drug shop	24 (6.5)	43 (11.2)		22 (6.0)	37 (9.7)	
Ordinary shop/weekly markets	7 (1.9)	18 (4.7)		13 (3.5)	27 (7.1)	
Traditional birth attendants	14 (3.8)	39 (10.2)		13 (3.5)	12 (3.1)	
Family planning clinics	30 (8.1)	110 (28.6)		28 (7.6)	66 (17.2)	
Drug/medicine vendors	22 (6.0)	35 (9.1)		15 (4.1)	33 (8.6)	
Other	7 (1.9)	14 (3.7)		5 (1.4)	12 (3.1)	
**Decision to use FP**						
Mainly mine (participant)	57 (38.0)	77 (37.4)	0.92	60 (43.8)	83 (46.9)	0.37
Mainly spouse/partner	7 (4.7)	8 (3.9)		13 (9.5)	8 (4.5)	
Joint decision	86 (57.3)	121 (58.7)		63 (46.0)	85 (48.0)	
Other	0 (0.0)	0 (0.0)		1 (0.7)	1 (0.6)	
**Discussion of FP with spouse**						
Never	31 (20.7)	21 (10.2)	0.04	26 (19.0)	26 (14.7)	0.76
Sometimes	54 (36.0)	71 (34.5)		55 (40.1)	74 (41.8)	
Often	35 (23.3)	56 (27.2)		24 (17.5)	35 (19.8)	
Always	30 (20.0)	58 (28.1)		32 (23.4)	42 (23.7)	
**Condom use in past 12 months**						
Sometimes/Inconsistent	189 (52.4)	169 (46.4)	0.11	185 (53.8)	156 (44.1)	0.01
Always	172 (47.6)	195 (53.6)		159 (46.2)	198 (55.9)	

### HIV and syphilis prevalence and incidence

The overall HIV prevalence was 14.6% (12.6% in the intervention and 16.7% in the control arm) while the overall Syphilis prevalence was 7.6% (8.4% in the intervention and 6.9% in the control arm) as shown in Table 7. The HIV incidence after 12 months of follow up was 2.7 per 100 PYAR (3.0 per 100 PYAR in the intervention and 2.4 per 100 PYAR in the control arm) while the syphilis incidence was 3.9 per 100 PYAR (2.4 per 100 PYAR in the intervention and 4.7 per 100 PYAR in the control arm). The difference in prevalence and incidence of HIV and syphilis between arms was not statistically significant.

**Table 7:** Prevalence and incidence of HIV and Syphilis infections in a trial that evaluated the effectiveness of enhanced FP education in Ugandan fishing communities

Arm	**HIV prevalence(*n*** **= 1004)**	**HIV incidence**			***p*-value**
	HIV Positive (*n* (%)	New Cases	PYAR	Incidence (95%CI)	
Overall	147 (14.6)	16	587.2	2.7 (1.7-4.5)	
Intervention	63 (12.6)	9	301.0	3.0 (1.6-5.7)	0.351
Control	84 (16.7)	7	286.2	2.4 (1.2-5.1)	
	**Syphilis Prevalence(*n*** **= 987)**^**a**^	**Syphilis incidence**			
	Syphilis positive *n* (%)	New Cases	PYAR	Incidence (95%CI)	
Overall	75 (7.6)	23	595.6	3.9 (2.6-5.8)	
Intervention	41 (8.4)	9	298.4	2.4 (1.6-5.8)	0.152
Control	34 (6.9)	14	297.2	4.7 (2.8-8.0)	

^a^17 had no syphilis results (11-intervention & 6 control arm)

## Discussion

This study evaluated the effectiveness of a simplified FP education handout on FP knowledge and use as compared with FP counselling which is routinely done among resident fisher-folk in their reproductive age. Both methods improved FP uptake and knowledge, however the intervention arm with an education handout demonstrated significant improvement over the current standard of care. The handout comprised counselling material with short phrases and pictures and it was given to participants to keep. After a follow-up duration of 12 months, the simplified FP education handout was 47% more effective in increasing FP knowledge than FP counselling which is routinely done. Studies in Uganda have evaluated changes in knowledge, uptake and sources of FP methods [14, 32, 33] but none assessed the content and quality of knowledge participants had. In our study, besides determining FP awareness and sources of information, we determined FP knowledge among participants. Although being aware of FP methods may be good, it is not enough. Good knowledge of the FP methods, how and when they should be used, and their side effects is important in choosing the right method to use.

In the current study, FP awareness was almost universal at baseline with pills, injectable hormones, condoms and implants being more popularly known than other FP methods (e.g., spermicides, periodic abstinence, calendar method, IUD, tubal ligation, vasectomy, rhythm/withdraw method, diaphragm, dermal patch). FP awareness elsewhere in the country has also been shown to be high, likely due to past efforts by the Ministry of Health and other implementing partners to sensitize the public about contraception [14, 33, 34]. There is currently a paucity of data on actual FP knowledge countrywide which demonstrates a gap in assessing the effectiveness of the sensitization efforts. We observed that FP knowledge in this subpopulation was poor regardless of the high FP awareness. Fishing communities tend to be remote, hard-to-reach villages, making health service provision challenging. This could impact knowledge and comprehension of health-related issues. In most fishing communities, there are few FP options available, and as such, the fisher-folk FP knowledge is limited to those methods available. It is likely that improving access of different FP options could yield better FP knowledge.

Another challenge is that the medium used to relay FP information to individuals in rural settings (lectures or counselling) tends to be similar to what is used for more affluent urban residents. Understanding medical information may be difficult for communities with low education levels, so formal counselling may not appeal to such communities. Just as it has been evidenced in this and other studies, most residents in fishing communities attained only up to primary education level. Their comprehension and interpretation of information may be poor. Understanding FP concepts may be difficult for people with low education levels and their knowledge is likely to be poor. This is supported by a study that assessed the effect of literacy on FP practices among married women in rural south India [22]. In this study, participants with high education levels were more likely to get higher knowledge scores as compared to those with lower education levels. We also previously noted a relationship between reported education levels and good knowledge [15]. Using communication media or innovative strategies that suit the education status of a given setting might be a cornerstone for improving comprehension. Innovative education strategies have been demonstrated to improve comprehension and eventual use of health services in some remote settings [35, 36].

Good FP knowledge is necessary in making informed decisions and using contraception correctly. In the current study, the intervention yielded higher FP knowledge scores compared to routine FP counselling. Education interventions that suit the social contexts of the local setting may be more suitable to facilitate behaviour change leading to attainment of reproductive health goals. Using reading material with short phrases and pictures to educate people about mechanisms of action of FP methods, their side effects and how they can be managed may appeal to populations that are characterized by low literacy levels. But given the complexity of some of the methods and the different ethnic backgrounds of the residents, a combination of different education interventions may yield even better results. Elsewhere, a systematic review which assessed the impact of contraceptive education on knowledge and decision making demonstrated that a range of education interventions increased the quality of FP knowledge [37]. In another systematic review on community education and engagement in FP, community education using traditional modalities had a positive impact on FP knowledge [38].

As has been observed elsewhere, the short acting reversible methods were used more than the long-acting reversible methods and the permanent methods [39]. We attribute this to the fact that most of the study participants belonged to the young age group (15-29 years) which may desire fertility or fertility control in short intervals. The lack of trained personnel and health facilities to offer the long acting or permanent methods could be another reason. Each study community has one government health facility and most residents get their FP services from government health facilities. Constructing more government health facilities or better stocking of existing facilities and deployment of trained health personnel need to be prioritized in these communities. Sensitization about benefits of longer fertility intervals when long-acting reversible methods are used is important. Increasing awareness of the FP side effects and how these can be managed may reduce the number that shuns FP.

Unmet need for modern FP, remains a big challenge in East Africa [40]. In Uganda, 40% married women and almost half of sexually active women of reproductive age have an unmet need and unsatisfied demand for FP [41]. In this study, we observed a 53% unmet need for FP among sexually active women. This constituted women who wanted to postpone their next birth for 2 or more years or those who wanted to stop childbearing altogether but were not using a contraceptive method. A 16% absolute increase in FP use in the intervention arm suggests limited contraceptive information as one of the factors contributing to the high unmet need in the fishing communities. As countries move towards achieving universal health coverage for FP, it is important to understand the cause of unmet need in different settings and how it may be leveraged to meet country specific FP goals. Improving the female fisher-folk’s contraceptive knowledge could be a critical strategy for reducing their unmet need.

The knowledge people have may influence attitudes, perceptions and FP practices. Different education strategies may impact knowledge differently depending on how and where they are employed. Elsewhere, community education and engagement in FP while using mass media, print or mail, web-based, text messaging, and interpersonal interventions have been found to have a positive impact on FP uptake [38]. A field based contraception education program improved FP use in another setting [42]. A study that was conducted in Kenya concluded that interventions which adapt to indigenous backgrounds can be acceptable to communities and are associated with significant changes in behaviour [43]. In the current study, the intervention was designed to suit the social context of the population while considering their literacy levels. We also observed that the intervention improved discussion of FP with spouses. It has been evidenced that better reproductive outcomes can be achieved when spouses discuss reproductive health matters [44–46]. Discussion of reproductive health issues such as FP by spouses or sexual partners could alleviate stigma and improve uptake.

Given the high sexual activity and multiple sexual partnerships in the fishing communities, STIs are common. We observed that the incidence and prevalence of HIV and Syphilis in this population were higher than the general population [8]. Other studies that were conducted in this population also reported higher HIV or syphilis infection rates in fishing communities compared to the general population highlighting a need for continuous sex and HIV/STI education to enable behavioural change [28–30]. We noted also that condom use by participants was low compared to that in the general population [47]. Condom use ought to be promoted in such communities with high sexual activity because they can be used for contraception and prevention of STIs. However, if not used consistently and correctly, condoms could be less effective.

### Study limitations

A curriculum-based FP education program preferably one that is validated and proven to be effective in similar populations would have yielded more reliable findings but none was available. Nevertheless, staff were trained on how to use the study tool and the tool was piloted in a non-study community prior its use. Generalizability of the study findings may be limited because the study sites were purposively selected. However, both study sites are large fishing villages and their location is such that one is a landing site and the other an island facilitating comparisons across the diverse range of fishing communities around Lake Victoria. Randomization minimized confounding and thus increased the internal validity of the study as we note that the two study arms were similar after randomization. The follow up phase may have been too short to produce significant changes in behaviour in regard to long-acting reversible methods such as implants and intra-uterine devices. A longer follow-up study would be recommended for assessing IUDs as these could not be assessed during the 6 monthly visits. Significant differences in HIV and syphilis infection rates across study arms could not be demonstrated given the short follow-up duration and limited sample size. Despite its limitations, this study provides empirical data that could inform future research. Data from this research can guide policy change and action regarding FP education in remote settings like fishing communities in Uganda.

### Interpretation

Enhanced FP education using a simplified FP handout that was based on the WHO Family Planning guidance tool was more effective in increasing FP knowledge and improving FP use when compared to routine FP counselling for people residing in fishing communities. We recommend innovative education tools to optimize FP knowledge and uptake for people residing in poor settings where literacy levels are low. However, due to the high cost incurred during designing of the tool, triangulated research may be necessary to provide a stronger evidence base for using such a tool in bigger populations. A qualitative assessment of participant perceptions of the study tool would also be beneficial. In view of the persistent high HIV and Syphilis infection rates that were evidenced in this sub-population, these communities should be targeted for provision of STI biomedical prevention interventions and related research. Finally we recommend continuous sexual health education and promotion of safe sex habits in these communities to ensure good health and the wellbeing of the fisher folk.

## Data Availability

Participant data was collected after provision of written informed consent under the prerequisite of strict participant confidentiality. The datasets used and/or analysed during the current study together with the Protocol are available from the corresponding author on reasonable request. A full data set containing the data supporting the study findings in this article can also be obtained from the Program Data Manager, by email to: tnakaweesa@iavi.or.ug or information@iavi.or.ug

## Abbreviations

CPR: Contraceptive Prevalence Rate
MoH: Ministry of Health
STI: Sexually Transmitted Infection
UNCST: Uganda National Council for Science and Technology
UVRI-REC: Uganda Virus Research Institute Research Ethics Committee
